# Giant obscurins regulate the PI3K cascade in breast epithelial cells via direct binding to the PI3K/p85 regulatory subunit

**DOI:** 10.18632/oncotarget.9985

**Published:** 2016-06-13

**Authors:** Marey Shriver, Saravanakumar Marimuthu, Colin Paul, Janelle Geist, Tessa Seale, Konstantinos Konstantopoulos, Aikaterini Kontrogianni-Konstantopoulos

**Affiliations:** ^1^ Department of Biochemistry and Molecular Biology, University of Maryland School of Medicine, Baltimore, MD 21201, USA; ^2^ Department of Chemical and Biomolecular Engineering, Johns Hopkins University, Baltimore, MD 21218, USA; ^3^ Johns Hopkins Institute for NanoBioTechnology, Johns Hopkins University, Baltimore, MD 21218, USA; ^4^ Johns Hopkins Physical Sciences-Oncology Center, Johns Hopkins University, Baltimore, MD 21218, USA; ^5^ University of Maryland School of Medicine, Marlene and Stewart Greenebaum National Cancer Institute Cancer Center, Baltimore, MD 21201, USA

**Keywords:** obscurin, PI3K, PI3K inhibitors, Akt, breast cancer

## Abstract

Obscurins are a family of giant cytoskeletal proteins, originally identified in striated muscles where they have structural and regulatory roles. We recently showed that obscurins are abundantly expressed in normal breast epithelial cells where they play tumor and metastasis suppressing roles, but are nearly lost from advanced stage breast cancer biopsies. Consistent with this, loss of giant obscurins from breast epithelial cells results in enhanced survival and growth, epithelial to mesenchymal transition (EMT), and increased cell migration and invasion *in vitro* and *in vivo*. In the current study, we demonstrate that loss of giant obscurins from breast epithelial cells is associated with significantly increased phosphorylation and subsequent activation of the PI3K signaling cascade, including activation of AKT, a key regulator of tumorigenesis and metastasis. Pharmacological and molecular inhibition of the PI3K pathway in obscurin-depleted breast epithelial cells results in reversal of EMT, (re)formation of cell-cell junctions, diminished mammosphere formation, and decreased cell migration and invasion. Co-immunoprecipitation, pull-down, and surface plasmon resonance assays revealed that obscurins are in a complex with the PI3K/p85 regulatory subunit, and that their association is direct and mediated by the obscurin-PH domain and the PI3K/p85-SH3 domain with a KD of ∼50 nM. We therefore postulate that giant obscurins act upstream of the PI3K cascade in normal breast epithelial cells, regulating its activation through binding to the PI3K/p85 regulatory subunit.

## INTRODUCTION

Obscurins are a family of giant, cytoskeletal proteins originally identified in striated muscles, where they play important roles in their structural organization and contractile activity [1, 2]. In humans, the *OBSCN* gene, encoding obscurins, spans 150 kb on chromosome 1q42 and gives rise to several isoforms through alternative splicing [3, 4]. The prototypical obscurin, obscurin-A, is ∼720 kDa and contains multiple signaling and adhesion domains arranged in tandem. The NH_2_-terminus and middle portion of obscurin-A contain repetitive immunoglobulin (Ig) and fibronectin-III (Fn-III) domains, while the COOH-terminus consists of several signaling domains, including an IQ motif, a src homology 3 (SH3) domain, a Rho-guanine nucleotide exchange factor (Rho-GEF), and a Pleckstrin Homology (PH) domain followed by a ∼400 amino acids long segment that contains ankyrin binding sites [5, 6]. The *OBSCN* gene gives rise to another large isoform, obscurin-B or giant Myosin Light Chain Kinase (MLCK), which has a molecular mass of ∼870 kDa. Two active serine/threonine kinase domains that belong to the MLCK subfamily are present in the extreme COOH-terminus of obscurin-B, which replace the ∼400 amino acids long COOH-terminus of obscurin-A [4, 7]. The two serine/threonine kinases may also be expressed independently as smaller isoforms, containing one (∼55 kDa) or both (∼145 kDa) domains.

Recent work from our laboratory has demonstrated that giant obscurins are abundantly expressed in normal breast epithelium, where they primarily localize at cell-cell junctions [8]. Their expression levels and subcellular localization, however, are altered in advanced stage human breast cancer biopsies [9]. Specifically, breast cancer biopsies of grade-2 or higher exhibit dramatically reduced levels of giant obscurins, while residual proteins concentrate in large cytoplasmic puncta [9]. Obscurin-depleted non-tumorigenic breast epithelial MCF10A cells exhibit a growth advantage under anchorage-independent conditions, form mammospheres enriched with markers of stemness, extend microtentacles, and undergo epithelial to mesenchymal transition (EMT) resulting in disruption of adherens junctions, and enhanced motility and invasion *in vitro* [9, 10]. Consistent with these major alterations, depletion of giant obscurins from MCF10A cells expressing an active form of the K-Ras oncogene results in primary and metastatic tumor formation in subcutaneous and lung metastasis *in vivo* models, respectively [9]. Taken together, these findings indicate that giant obscurins act as tumor and metastasis suppressors in normal breast epithelium. Conversely, their loss potentiates tumorigenicity and induces metastasis.

In the present study, we sought to mechanistically understand how loss of giant obscurins leads to the aforementioned phenotypic and functional manifestations in breast epithelial cells. We found that down-regulation of giant obscurins in MCF10A breast epithelial cells leads to dramatic up-regulation of the Phosphoinositide-3 kinase (PI3K) signaling cascade. Notably, the PI3K pathway is altered in > 30% of invasive breast carcinoma cases (http://www.mycancergenome.org/content/disease/breast-cancer/; Targeting PI3K in breast cancer). Our data reveal that pharmacological or molecular inhibition of the PI3K pathway results in reversal of EMT and suppression of the growth, motility, and invasion capabilities of obscurin-depleted MCF10A cells. Thus, loss of giant obscurins from breast epithelial cells induces a tumorigenic and metastatic phenotype, at least in part, via up-regulation of the PI3K pathway. This is corroborated by our biochemical studies demonstrating for the first time that in normal breast epithelial cells giant obscurins and PI3K interact directly at the level of the cell membrane. Collectively, our findings indicate that giant obscurins act upstream of the PI3K pathway in breast epithelial cells contributing to its regulation.

## RESULTS

### Downregulation of giant obscurins in normal breast epithelial cells results in upregulation of the PI3K pathway

We previously generated stable MCF10A obscurin-knockdown cell lines using shRNAs targeting sequences within the common NH_2_-terminus and middle portion of giant obscurins A and B [8, 9]. Obscurin-knockdown MCF10A cells undergo major cytoskeletal remodeling leading to increased tumorigenicity, motility and invasion both *in vitro* and *in vivo* [8, 9]. However, the molecular alterations accompanying obscurins’ loss from breast epithelial cells have yet to be delineated.

**Figure 1:**
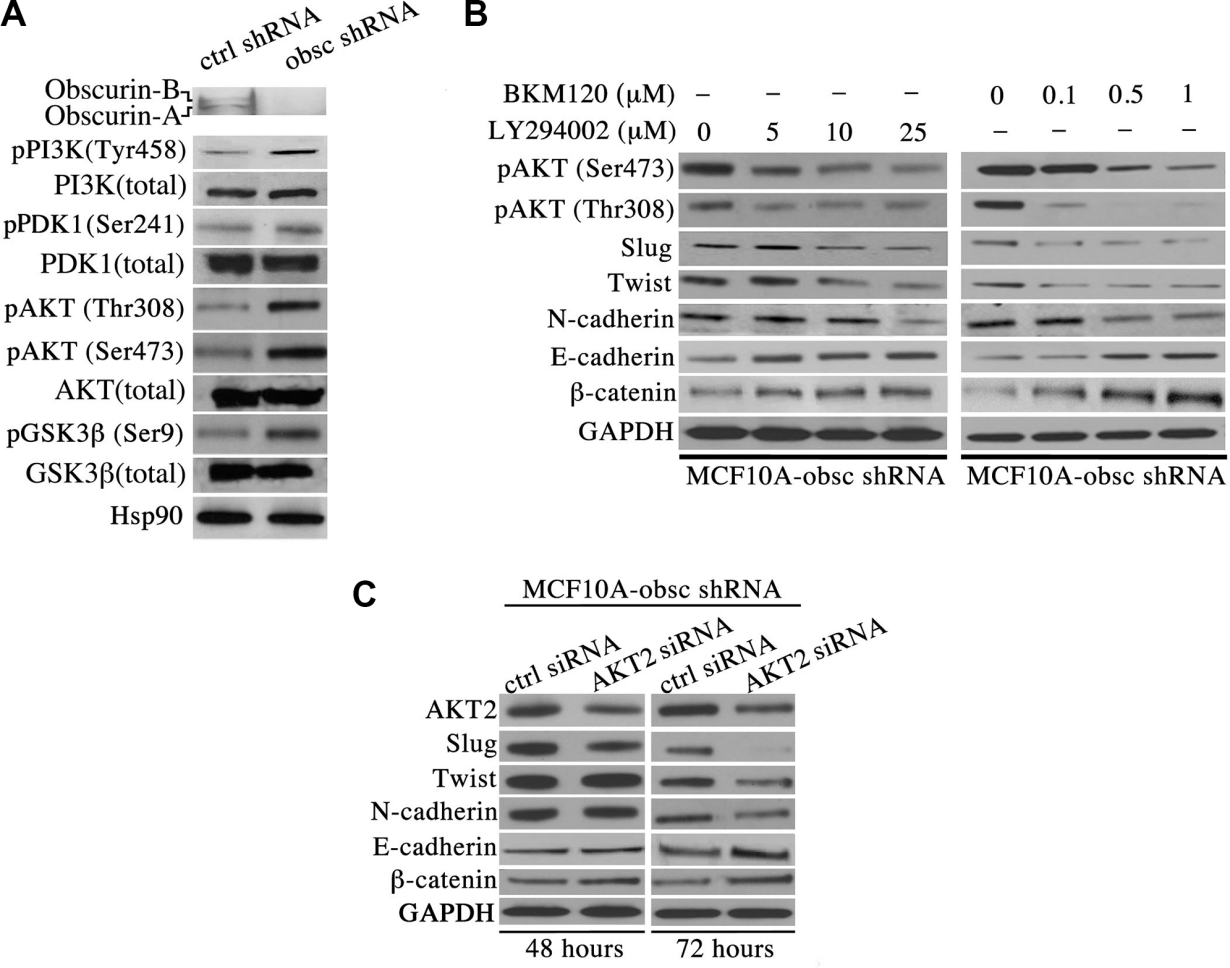
Activation of the PI3K pathway in obscurin-knockdown MCF10A cells underlies the acquisition of EMT (**A**) Western blot analysis of proteins associated with the PI3K cascade indicates that obscurin-knockdown MCF10A cells exhibit increased levels of phosphorylated forms of PI3K (at tyrosine-458, 1.5-fold), PDK1 (at serine-241, 1.2-fold), AKT (at threonine-308, 3.7-fold, and serine-473, 2.5-fold) and GSK3β (at serine-9, 2.6-fold), compared to control cells stably expressing scramble shRNA. Notably, the total levels of PI3K, PDK1, AKT and GSK3β are unaltered in obscurin-knockdown cells. (**B**) Treatment of obscurin-knockdown MCF10A cells with various concentrations of the PI3K inhibitors LY294002 (0–25 μM) and BKM120 (0–1 μM) results in a dose-dependent decrease in the phosphorylation levels of AKT at both serine-473 (0.4-fold at 25 μM LY294002 & 0.3-fold at 1 μM BKM120) and threonine-308 (0.4-fold at 25 μM LY294002 & 0.1-fold at 1 μM BKM120), compared to cells treated with vehicle DMSO. More importantly, inhibitor-treated obscurin-knockdown cells exhibit a dose-dependent decrease in the expression levels of the mesenchymal protein N-cadherin (0.3-fold at 25 μM LY294002 & 0.5-fold at 1 μM BKM120) and the transcriptional regulators Slug (0.6-fold at 25 μM LY294002 & 0.3-fold at 1 μM BKM120) and Twist (0.4-fold at 25 μM LY294002 & 0.5-fold at 1 μM BKM120), and a concomitant increase in the expression levels of the epithelial proteins E-cadherin (1.8-fold at 25 μM LY294002 & 2.1-fold at 1 μM BKM120) and β-catenin (1.3-fold at 25 μM LY294002 & 5-fold at 1 μM BKM120). (**C**) Stable clones of obscurin-knockdown MCF10A cells treated with a siRNA specifically targeting AKT2 exhibited 0.7- and 0.6-fold decrease in the expression levels of AKT2 at 48 and 72 hours post-transfection, respectively. Similarly to PI3K inhibitor treatment, down-regulation of AKT2 results in markedly decreased levels of the mesenchymal proteins Slug (0.1-fold), Twist (0.6-fold) and N-cadherin (0.5-fold), and increased levels of the epithelial proteins E-cadherin (2.2-fold) and β-catenin (1.8-fold) at 72 hours post-treatment.

Mounting evidence suggests the pivotal role of the PI3K signaling cascade in regulating multiple processes during breast cancer formation and metastasis, including cell growth, migration, invasion and distant colonization [11]. We therefore interrogated the expression levels and phosphorylation state of major components of the PI3K pathway in MCF10A obscurin-knockdown cells. Immunoblotting analysis revealed a significant increase in the levels of the phosphorylated forms of major components of the PI3K pathway in MCF10A obscurin-knockdown cells compared to controls (Figure 1A). In particular, we detected a considerable increase in the amounts of phosphorylated PI3K at tyrosine-458, a phospho-site that has been reported to track with the activation levels of the enzyme [12], PDK1, a downstream target of PI3K, at serine-241 that renders the enzyme catalytically active [13, 14], AKT, a direct target of PDK1, at threonine-308 and serine-473 indicating its maximal activation [15, 16], and GSK3β, a downstream target of AKT, at serine-9 leading to its inactivation that promotes cell cycle progression through stabilization of cyclin D1 [17]. Thus, depletion of giant obscurins from breast epithelial cells leads to increased phosphorylation and thus aberrant activation of the PI3K signaling cascade.

### Inhibition of PI3K signaling in obscurin-knockdown MCF10A cells reverses epithelial to mesenchymal transition

We have previously demonstrated that depletion of giant obscurins from MCF10A cells results in epithelial to mesenchymal transition (EMT) [9]. To examine whether activation of the PI3K pathway in obscurin-knockdown MCF10A cells underlies EMT, we used two well-known chemical inhibitors of PI3K, LY294002 and BKM120. Both LY294002 and BKM120 inhibit the catalytic subunit p110 of PI3K through direct binding and competition at its ATP-binding site.

To verify the effectiveness of the two inhibitors in suppressing the PI3K pathway, we first examined whether the phosphorylation levels of AKT were reduced following treatment. Indeed, treatment of obscurin-knockdown cells with varying concentrations of the LY294002 (0–25 μM) and the BKM120 (0–1 μM) inhibitors resulted in a dose-dependent decrease of the phosphorylation levels of AKT at both serine-473 and threonine-308 (Figure 1B), indicating that both inhibitors can effectively suppress the activation of the PI3K cascade. We then evaluated whether inhibition of PI3K reverses EMT in obscurin-knockdown cells. Examination of the expression levels of major epithelial and mesenchymal proteins by immunoblotting revealed a dose-dependent decrease in the amounts of the mesenchymal transcription factors Slug and Twist, and their downstream target N-cadherin, and a concomitant increase in the amounts of the epithelial proteins E-cadherin and β-catenin (Figure 1B).

To further demonstrate that treatment of obscurin-knockdown MCF10A cells with either the LY294002 or the BKM120 inhibitor reverses EMT specifically through suppression of the PI3K pathway, we transiently down-regulated the expression of members of the AKT family using siRNA technology, and examined the expression levels of the same battery of mesenchymal and epithelial proteins 48 and 72 hours post-transfection. While down-regulation of AKT1 failed to exert any effect at either time point (data not shown), down-regulation of AKT2, which promotes breast tumor growth and metastasis [18], resulted in decreased amounts of Slug, Twist and N-cadherin, and increased amounts of E-cadherin and β-catenin, with a more pronounced effect at 72 hours (Figure 1C).

**Figure 2:**
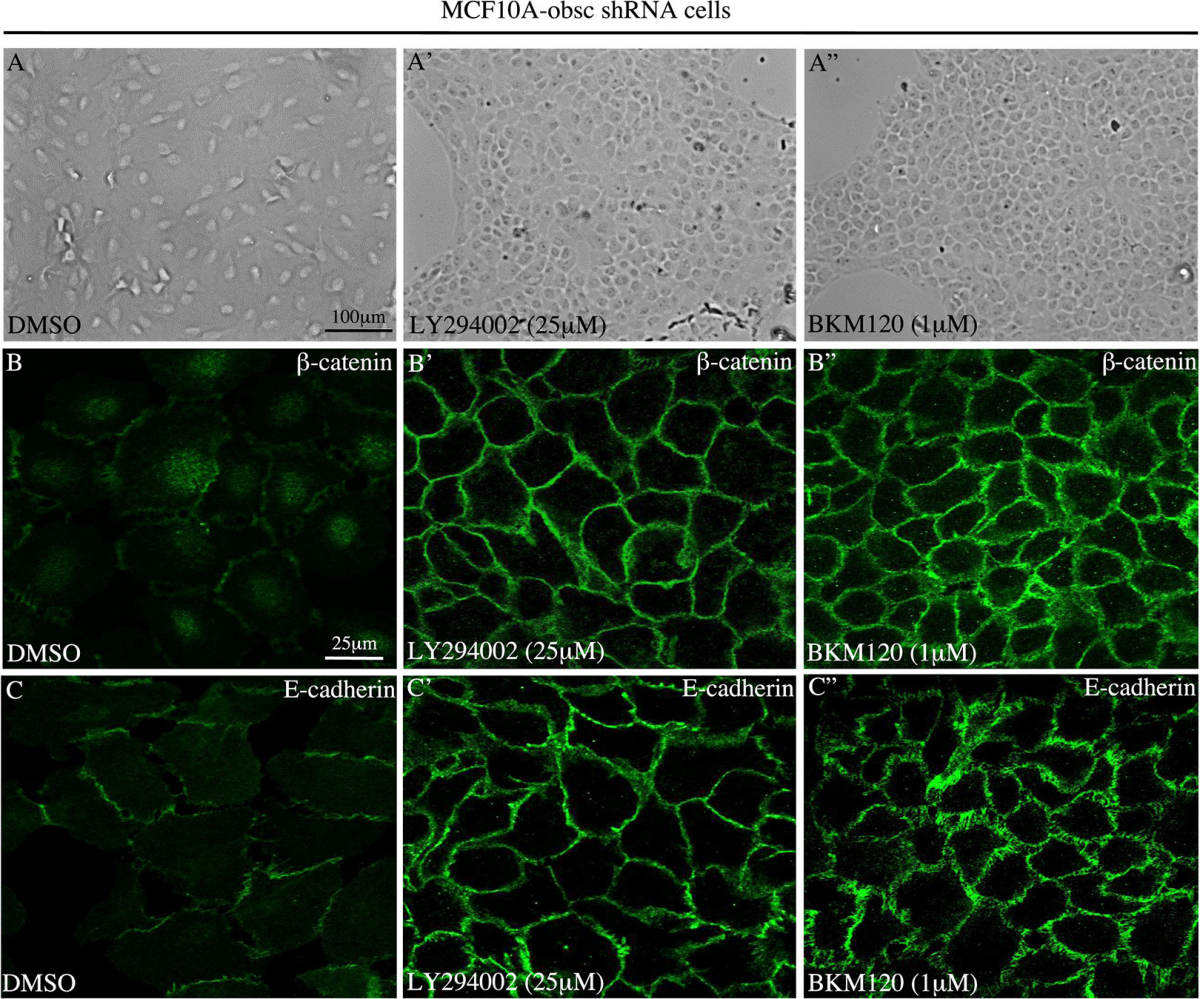
Treatment of MCF10A obscurin-knockdown cells with PI3K inhibitors restores the formation of cell-cell junctions (**A**–**A”**) Representative bright-field images of obscurin-knockdown MCF10A cells treated with DMSO vehicle or PI3K inhibitors. Cells treated with 25 μM LY294002 or 1 μM BKM120 lose their mesenchymal appearance, and instead acquire an epithelial morphology and form cell-cell junctions. The expression levels and membrane distribution of the epithelial markers β-catenin (**B**–**B”**) and E-cadherin (**C**–**C”**) are restored in MCF10A obscurin-knockdown cells treated with either PI3K inhibitor, as determined under confocal optics.

In agreement with these molecular alterations, evaluation of MCF10A obscurin-knockdown cells treated with either 25 μM of LY294002 or 1 μM of BKM120 using bright-field microscopy demonstrated that they (re)acquired an epithelial appearance and were able to form cell-cell junctions (Figure 2A–2A”). More importantly, examination of the subcellular distribution of the epithelial markers β-catenin (Figure 2B–2B”) and E-cadherin (Figure 2C–2C”) under confocal optics confirmed the increased expression of both proteins and revealed their accumulation at cell-cell contact sites, where they contribute to the formation and stabilization of adherens junctions.

Taken together, our findings indicate that blockade of the PI3K pathway in obscurin-depleted breast epithelial cells via pharmacological treatment or molecular means effectively reverses EMT.

### Suppression of the PI3K pathway diminishes the growth, motility and invasion potential of obscurin-depleted breast epithelial cells

**Figure 3:**
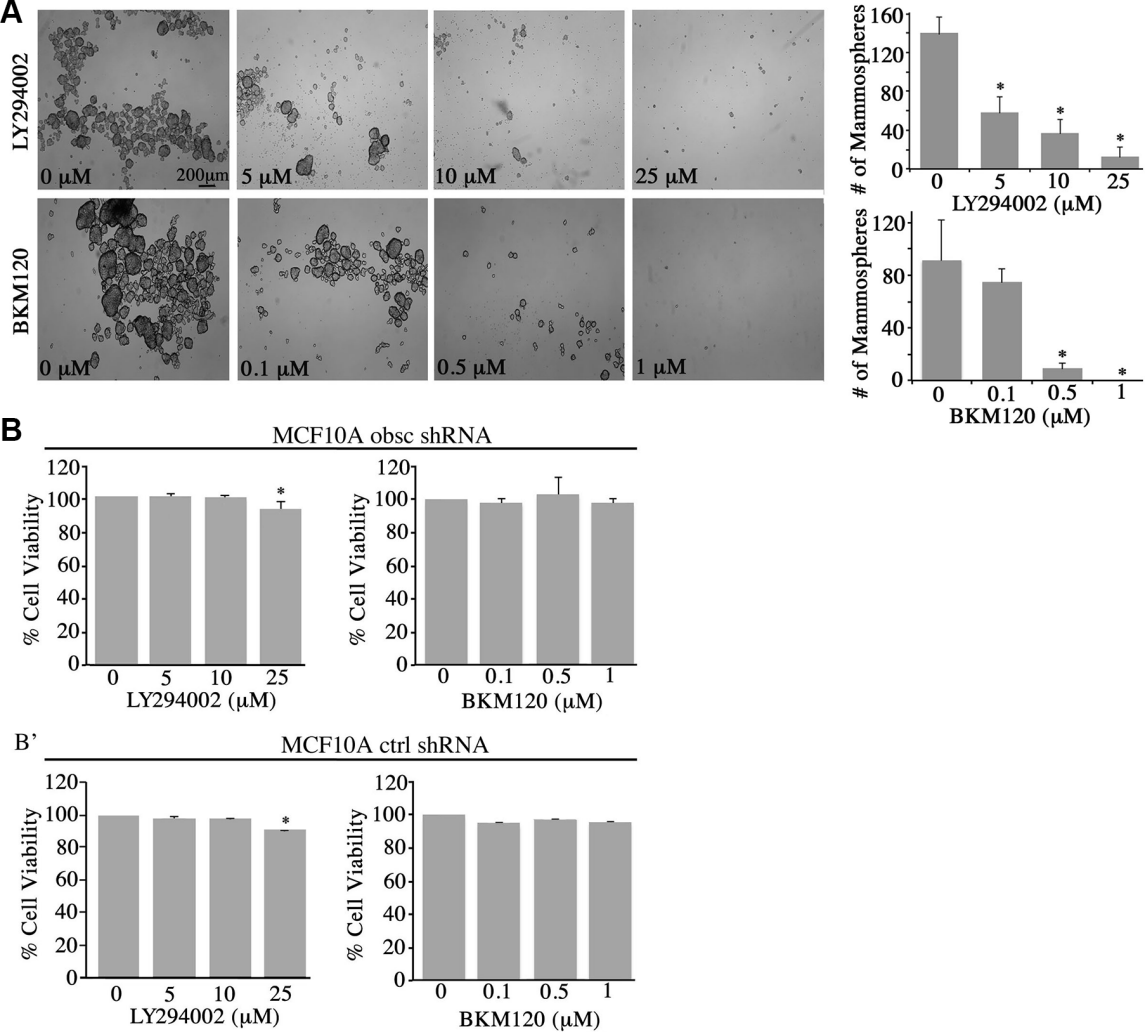
Inhibition of the PI3K pathway in obscurin-knockdown MCF10A cells decreases mammosphere formation (**A**) Representative bright-field images of obscurin-knockdown MCF10A cells treated with different concentrations of LY294002 (0–25 μM) and BKM120 (0–1 μM) inhibitors. Inhibitor-treated cells formed less and smaller mammospheres, compared to control cells treated with vehicle DMSO, when grown in ultra-low attachment conditions; scale bar 200 μm. Quantification of the number of mammospheres ≥ 100 μm formed by obscurin-knockdown MCF10A cells treated with LY294002 (0–25 μM) or BKM120 (0–1 μM); *n* = 3, error bars = SD, *P < 0.03; *t*-test. (**B**–**B’**) Evaluation of cell viability via quantification of percent (%) reduction of AlamarBlue following treatment of adherent MCF10A obscurin-knockdown (B) and scramble control (B’) cells with different concentrations of LY294002 or BKM120 for 24 h. No significant changes were observed between vehicle DMSO or either inhibitor treatment in the reduction of AlamarBlue reagent, with the exception of 25 μM LY294002 where we measured a small, but significance, decrease (∼5%) in cell viability; *n* = 3, error bars = SD, *P < 0.05; *t*-test.

**Figure 4:**
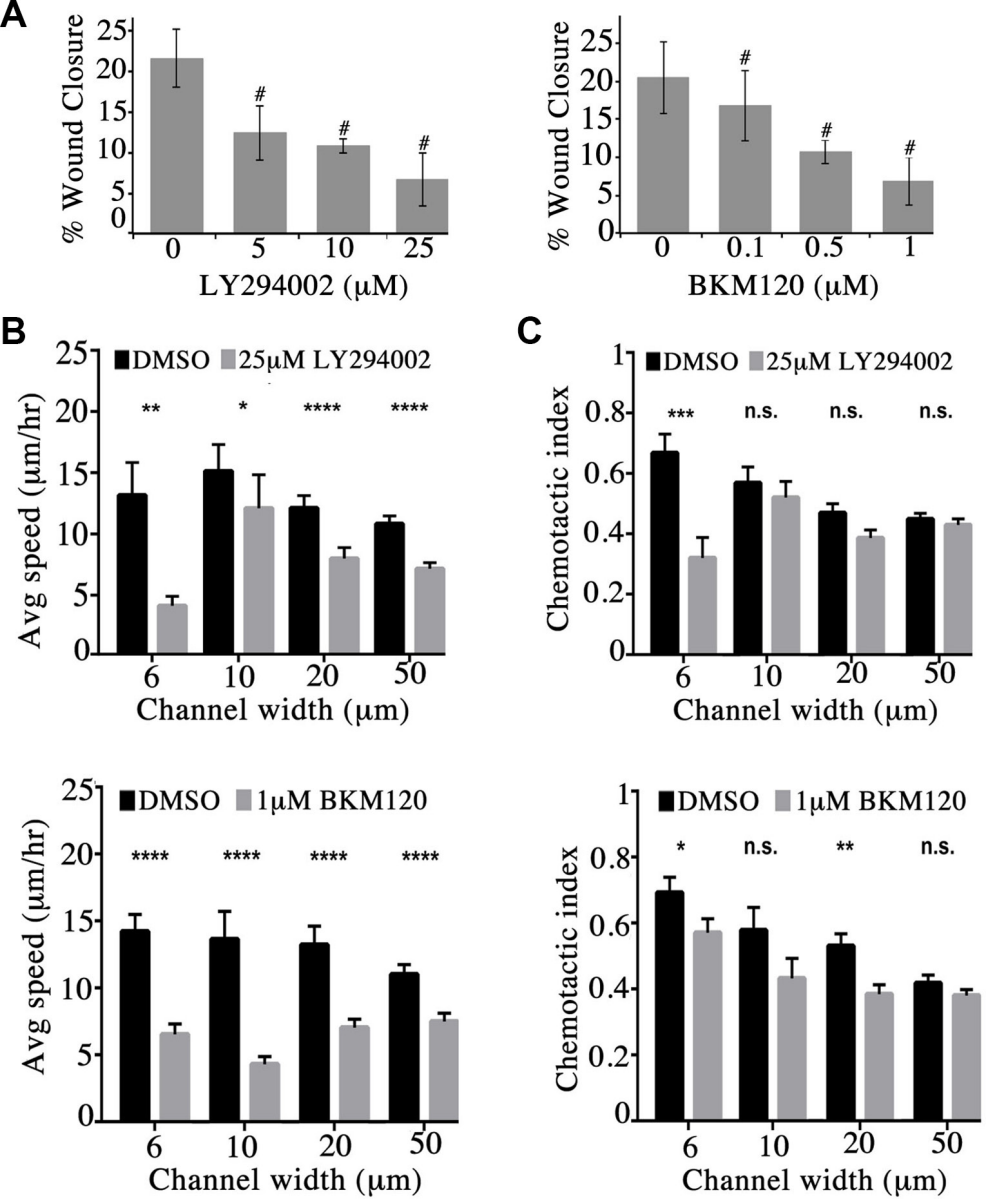
Obscurin-knockdown MCF10A cells exhibit reduced migration upon inhibition of the PI3K signaling cascade (**A**) Quantification of percent (%) wound closure of obscurin-knockdown MCF10A cells 6 h post-wounding; *n* = 3, error bars = SD, ^#^*P* < 0.03; *t*-test. (**B–C**) Cell speed and chemotactic index of obscurin-knockdown MCF10A cells treated with vehicle DMSO, 25 μM LY294002 or 1 μM BKM120 inhibitor as a function of microchannel width (6–50 μm). Inhibitor treated cells exhibit significantly decreased speed at both narrow (6 μm and 10 μm) and wide (20 μm and 50 μm) channels; *n* ≥ 5, at least 18 cells were analyzed per condition, error bars = SEM, *P < 0.05, **P < 0.01, ***P < 0.001, ****P < 0.0001; Mann-Whitney; ns: not significant.

**Figure 5:**
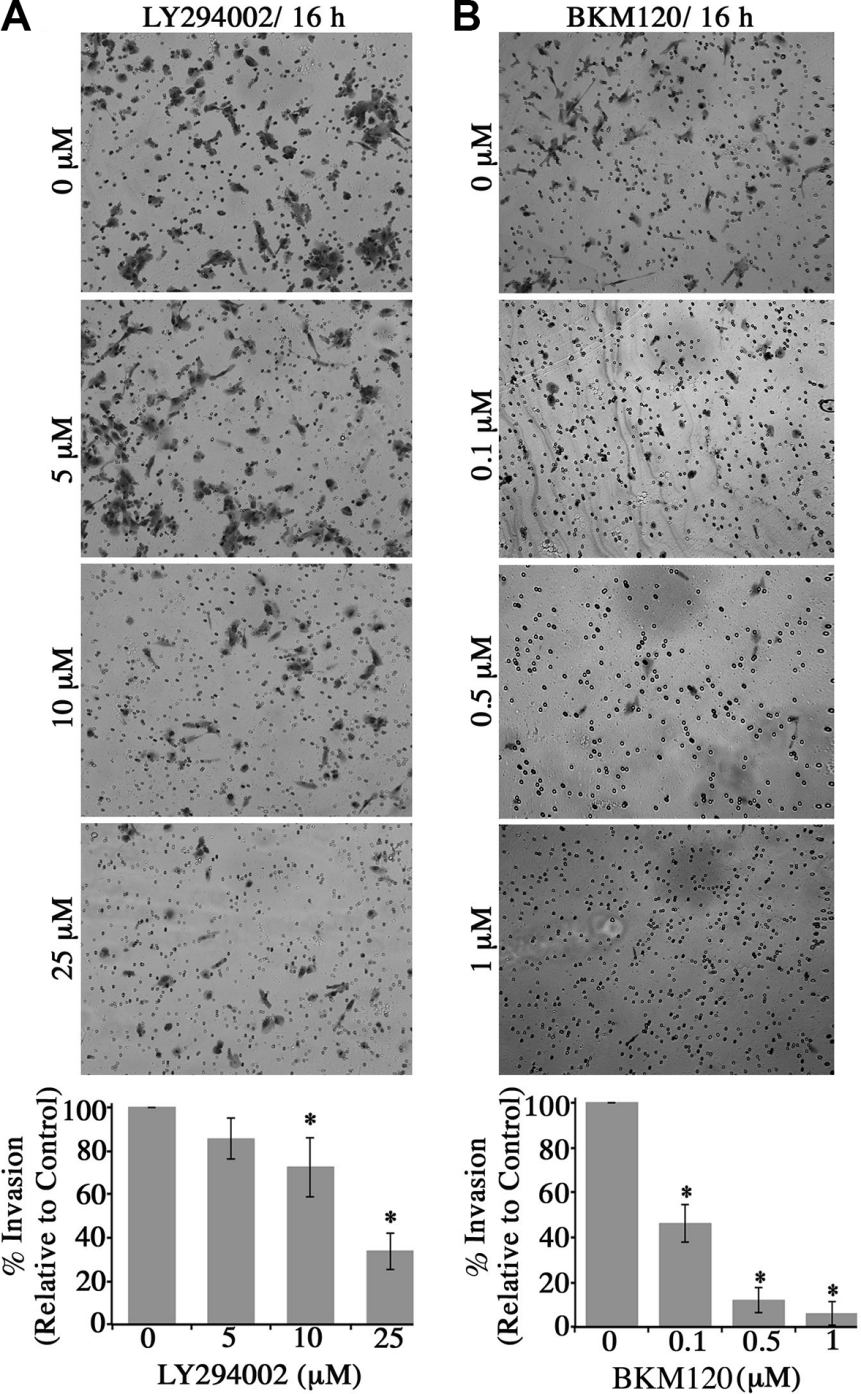
Blockade of the PI3K signaling in obscurin-knockdown MCF10A cells decreases their invasive capabilities through matrigel-coated chambers Stable clones of MCF10A cells expressing obscurin shRNA were added to a matrigel-coated chamber in the presence of different concentrations of (**A**) LY294002 (0–25 μM) or (**B**) BKM120 (0–1 μM), and allowed to invade for 16 h. Invasive cells were visualized via staining with crystal violet dye. Treatment with either inhibitor markedly decreased the invasive capabilities of obscurin-knockdown MCF10A cells in a dose-dependent manner. Quantification of the % invasion of inhibitor-treated relative to DMSO-treated obscurin-knockdown cells, which was arbitrarily set to 100%; *n* = 3, error bars = SD, *P < 0.03; *t*-test.

The ability of epithelial cancer cells to undergo EMT provides them with a growth advantage and increased motility and invasion capabilities [19]. Consistent with this, our previous studies demonstrated that obscurin-knockdown MCF10A cells form robust primary and secondary mammospheres (≥ 100 μm) enriched in stem cell markers under low attachment conditions, and display markedly increased tumorigenicity, motility and invasiveness *in vitro* and *in vivo* [9]. We therefore sought to examine if the enhanced tumorigenic, motile and invasive properties of the obscurin-knockdown MCF10A cells are due to up-regulation of the PI3K pathway. To address these questions, we treated stable clones of obscurin-knockdown MCF10A cells with different concentrations of the LY294002 (0–25 μM) and BKM120 (0–1 μM) PI3K inhibitors and examined their ability to survive and grow under low attachment conditions (Figure 3), migrate collectively and as single cells (Figure 4), and invade though artificial extracellular matrix (Figure 5).

Treatment with varying concentrations of LY294002 (0–25 μM) or BKM120 (0–1 μM) resulted in a marked decrease in the number and size of mammospheres formed by the obscurin-knockdown MCF10A cells (Figure 3A). Specifically, treatment with 5 μM and 10 μM of LY294002 resulted in 36% and 58% reduction in mammosphere formation, respectively, while treatment with 25 μM of LY294002 nearly abolished mammosphere formation. Similarly, treatment with 0.1 μM and 0.5 μM of BMK120 resulted in 18% and 89% reduction in mammosphere formation, respectively, while treatment with 1 μM BKM120 completely eliminated mammosphere formation. It is noteworthy that treatment of adherent MCF10A obscurin-knockdown or scramble control cells with either inhibitor did not affect their survival and growth, except in the case of 25 μM LY294002 where we observed a slight, yet significant, decrease (∼5%) in cell viability (Figure 3B–3B’). Thus, these results pinpoint the importance of active PI3K signaling in promoting survival and growth of obscurin-depleted breast epithelial cells under unfavorable (i.e. anchorage-independent) conditions.

We next examined if inhibition of the PI3K pathway in obscurin-knockdown MCF10A cells decreases their migratory ability. We therefore treated MCF10A obscurin-knockdown cells plated on collagen with different concentrations of the LY294002 (0–25 μM) and BKM120 (0–1 μM) inhibitors and examined their migratory potential in wound healing assays (Supplementary Figure S1). Inhibition of the PI3K pathway with either inhibitor resulted in a dramatic and dose-dependent decrease of the migratory capability of the obscurin-knockdown cell monolayer over a 6-hour time period. While obscurin-knockdown cells treated with DMSO vehicle exhibited ≥ 20% wound healing after 6 h, cells treated with 25 μM of LY294002 or 1 μM of BKM120 exhibited ∼6% wound healing (Figure 4A).

We also evaluated the ability of single obscurin-knockdown MCF10A cells treated with either 25 μM of LY294002 or 1 μM of BKM120 to migrate through collagen type I-coated microchannels of constant height (i.e. 10 μm) and varying widths (i.e. 6, 10, 20 and 50 μm) using a microfluidic-based migration chamber combined with live-cell phase-contrast imaging. Obscurin-knockdown cells treated with either inhibitor exhibited significantly reduced migration in both narrow (i.e. 6 and 10 μm) and wide (i.e. 20 and 50 μm) microchannels, as evidenced by their decreased migration speed compared to DMSO treated cells (Figure 4B, Supplementary Videos S1 and S2, and Supplementary Figure S2). Moreover, cells treated with either inhibitor relative to vehicle control exhibited lower chemotactic index (CI), which is a measure of cell migration persistence, and is calculated from the ratio of net cell displacement to the total distance traveled by the cell (Figure 4C). This decrease reached statistical significance in narrow channels (6 μm) for both inhibitors. Taken together, these data indicate that PI3K inhibition impairs single cell migration of obscurin-knockdown MCF10A cells primarily by affecting migration speed, with a secondary effect of decreasing cell directionality (particularly in highly confining, 6 μm, microchannels).

Lastly, we examined whether blockade of the PI3K cascade in obscurin-knockdown cells affected their invasive capability through a Matrigel-coated chamber (Figure 5). Similar to the wound healing and microchannel assays, treatment of obscurin-knockdown cells with varying concentrations of the LY294002 (0–25 μM; Figure 5A) and the BKM120 (0–1 μM; Figure 5B) inhibitor resulted in a significant, dose-dependent reduction in cell invasiveness, ranging from 15–60% (Figure 5A) and 50–95% (Figure 5B). Therefore, the increased activation of the PI3K pathway in obscurin-depleted breast epithelial cells is critical for their increased invasiveness through extracellular matrix and basement membranes.

### Giant obscurins interact directly with the p85 regulatory subunit of PI3K

**Figure 6:**
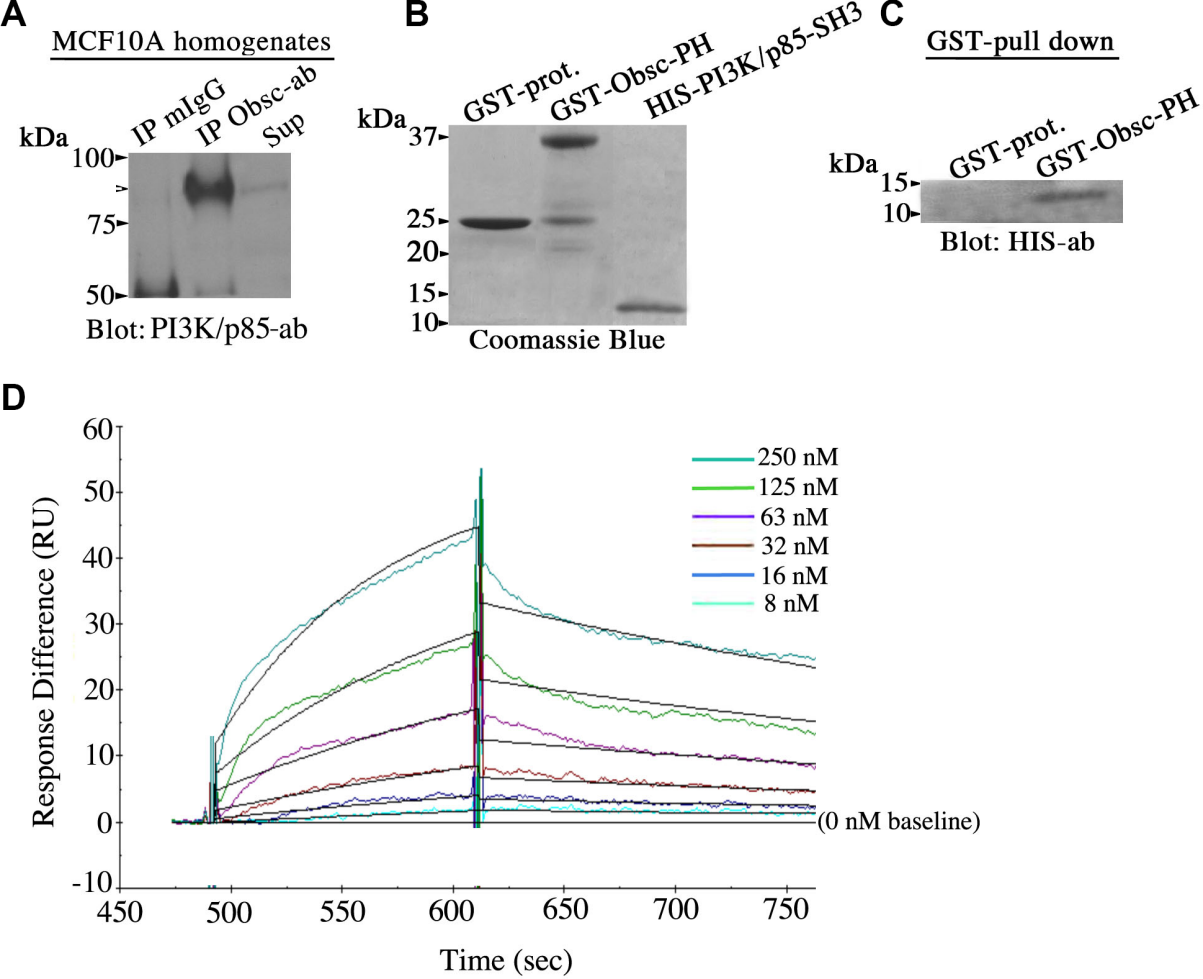
Giant obscurins directly interact with the PI3K/p85 regulatory subunit (**A**) Co-immunoprecipitation experiments using protein lysates prepared from MCF10A cells and control mouse IgG or antibodies to Ig1 of giant obscurins probed with a PI3K/p85 antibody. PI3K/p85 was readily detected in the immunoprecipitate fraction generated with the obscurin antibody (arrowhead), but not with the control mouse IgG; Sup: supernatant of the obscurin antibody immunoprecipitate fraction. (**B**) Coomassie blue stained gel showing bacterially expressed GST-protein (∼25 kDa), GST-obscurin-PH (∼37 kDa) and HIS-PI3K/p85-SH3 (∼10 kDa) after purification. (**C**) Equivalent amounts of control GST-protein and GST-obscurin-PH were incubated with 3 μg/ml of HIS-PI3K/p85-SH3. GST-obscurin-PH, but not GST-alone, is able to efficiently retain HIS-PI3K/p85-SH3, as shown by immunoblotting with antibodies to the HIS-tag. (**D**) Real time kinetics evaluation of the interaction between GST-obscurin-PH and HIS-PI3K/p85-SH3 using a Biacore 3000 surface plasmon resonance biosensor. A dissociation constant, K_D_, of ∼50 nM was calculated at equilibrium (Chi^2^ = 0.8).

To decipher how depletion of giant obscurins from breast epithelial cells leads to up-regulation of the PI3K cascade, we examined if giant obscurins and PI3K are direct binding partners. As a first step, we generated protein lysates from parental MCF10A cells that readily express obscurins and PI3K and performed co-immunoprecipitation assays using either antibodies to the extreme NH_2_-terminal immunoglobulin domain 1 (Ig1) of obscurins or control mouse IgG. The immunoprecipitate fractions were then tested for the presence of different components of the PI3K pathway via immunoblotting analysis. We found that the p85 regulatory component of PI3K was specifically and consistently present in the obscurin, but not the control, immunoprecipitate fraction (Figure 6A). We also examined the presence of additional components of the PI3K cascade in the obscurin immunoprecipitate fraction, including PDK1 and AKT, but we failed to detect them in our system (data not shown), which indicates the transient nature of their association with PI3K, and their lack of association with obscurins.

Giant obscurins contain a tandem array of signaling motifs in their extreme COOH-terminus, including a Pleckstrin Homology (PH) domain. It has been well-documented that PH domains are binding sites for lipids and/or other protein modules, and in particular for Src Homology 3 (SH3) domains [20, 21]. Importantly, the extreme NH_2_-terminus of the regulatory p85 subunit of PI3K contains an SH3 domain [22]. We therefore examined if giant obscurins interact with the PI3K/p85 subunit via the direct binding of their PH and SH3 domains, respectively. To test this, we generated recombinant obscurin-PH domain fused to GST and PI3K/p85-SH3 domain tagged with the HIS-moiety (Figure 6B), and performed a GST-pull down assay. GST-obscurin-PH, but not control GST-protein, was able to efficiently and specifically retain HIS-PI3K/p85-SH3 (Figure 6C). Moreover, to determine the strength of the interaction between the obscurin-PH and the PI3K/p85-SH3 domains, we performed real-time kinetics analysis using a Biacore 3000 surface plasmon resonance biosensor (Figure 6D). Quantification of the obtained sensogram data using BIA evaluation 3.1. software followed by fitting with the 1:1 Langmuir model yielded a binding affinity, K_D_, of ∼50 nM, which is indicative of strong, yet dynamic, binding between the obscurin-PH and the PI3K/p85-SH3 domains. Collectively, these findings indicate that giant obscurins and the p85 regulatory subunit of PI3K interact directly at the level of the plasma membrane where they both reside, and that their direct binding is mediated by their respective PH and SH3 domains.

## DISCUSSION

Deregulation of signaling pathways is a hallmark of cancer cells, and is intimately associated with abnormal growth under unfavorable conditions, and increased motility, invasiveness and colonization. We have recently demonstrated that the giant cytoskeletal proteins obscurins are abundantly expressed in normal breast epithelium where they preferentially concentrate at the plasma membrane [8, 9]. However, they are nearly lost from advanced grade (grade 2 or higher) human breast cancer biopsies [9]. Loss of giant obscurins from breast epithelial cells renders them highly tumorigenic and metastatic via induction of EMT that is associated with major cytoskeletal alterations [9]. We herein show that depletion of giant obscurins from MCF10A breast epithelial cells results in upregulation of the PI3K signaling pathway, as evidenced by the increased phosphorylation levels of major components of the cascade, including PI3K, PDK1, AKT and GSK3β. Inhibition of the PI3K pathway in obscurin-depleted MCF10A cells via pharmacological or molecular means reverses EMT, leading to substantially decreased growth, motility and invasiveness. Given the direct and strong interaction between obscurins and the PI3K/p85 regulatory subunit that our binding studies demonstrated, we postulate that loss of obscurins leads to conformational and/or molecular alterations of the PI3K/p85 subunit that render it unable to modulate the enzymatic activity of the PI3K/p110 catalytic subunit, thereby leading to its over-activation. Aberrant activation of the PI3K cascade, and its downstream target AKT, affects diverse (patho)physiological processes, including differentiation, growth, stemness, EMT, motility and invasiveness, which are intimately associated with increased tumorigenesis and metastasis [23].

The AKT family is composed of three isoforms, AKT1, AKT2 and AKT3 that are structurally homologous, but exhibit distinct functional properties [18]. Earlier studies have reported that overexpression of AKT1 inhibits breast epithelial cell migration and invasion via suppression of the ERK signaling pathway [24]. Conversely, silencing of AKT1 in non-tumorigenic MCF10A breast epithelial cells results in increased migration via induction of insulin-like growth factor (IGF) mediated cascades [25]. On the contrary, the levels of AKT3 mRNA, protein, and enzymatic activity are significantly increased in estrogen receptor negative (ER^−^) and triple negative (ER^−^, Progesterone Receptor negative, Her2/neu negative; ER^−^PR^−^Her2/neu^−^) breast cancers, promoting cell proliferation and tumor growth [26]. Similarly, amplification or overexpression of AKT2 has been identified in HER2/neu positive (Her2/neu^+^) breast cancers as well as ovarian, prostate and pancreatic cancers correlating with poor prognosis, and increased risk of relapse and metastatic tumor formation [27–30]. In agreement with these findings, overexpression of enzymatically inactive AKT2 in breast and ovarian cancer cells diminishes their motility and invasion capabilities *in vitro* and their ability to form tumors *in vivo,* whereas overexpression of full length AKT2 results in increased survival, migration and invasion *in vitro*, and formation of multiple adherent and non-adherent metastatic modules *in vivo* [31]. In line with these observations, our findings demonstrate that down-regulation of AKT2, but not AKT1, in obscurin-knockdown MCF10A cells results in diminished growth, migration and invasion.

A key mechanism underlying increased tumorigenicity and metastasis in cancer epithelial cells is the acquisition of a mesenchymal phenotype via EMT [19]. A prominent alteration that occurs during EMT is the concomitant loss of E-cadherin from adherens junctions and the increased expression of N-cadherin. This E-cadherin/N-cadherin switch is regulated by a number of transcription factors, including Slug, Snail and Twist; while all three repress the transcription of E-cadherin, Twist also induces the transcription of N-cadherin [32]. Earlier evidence has suggested that Slug, Snail and Twist may be direct targets of the PI3K/AKT cascade. In support of this notion, activation of the PI3K/AKT pathway in HER2-overexpressing MDA-MB-435 cancer cells leads to increased expression of Slug [33]. Similarly, activation of the PI3K/AKT cascade in melanoma, squamous carcinoma and breast cancer cell lines results in increased transcriptional activity of Twist and Snail [34–36]. We therefore postulate that the increased levels of Slug and Twist along with the E-cadherin/N-cadherin switch that takes place in the obscurin-knockdown cells during EMT is a direct result of the upregulation of the PI3K/AKT cascade.

Experimental evidence has highlighted the ability of cancer cells to evade anoikis and exhibit increased proliferation under non-adherent conditions, two properties that are associated with increased tumorigenicity [37]. Accordingly, enhanced activation of the PI3K/AKT pathway in breast cancer cells has been correlated with increased mammosphere and tumor formation *in vitro* and *in vivo,* respectively, via phosphorylation and activation of the master transcriptional factor NF-κB, which in turn regulates the expression of several genes involved in cell cycle progression and apoptosis [38, 39]. Transcriptional activation of NF-κB via the PI3K/AKT pathway also leads to up-regulation and increased production of matrix metalloproteinases (MMPs), which degrade the extracellular matrix and promote cell invasion [16, 40–42]. Moreover, activation of the PI3K/AKT cascade promotes cell motility by modulating the activity of major cytoskeletal proteins, such as girdin and filamin-A. Both girdin, an actin-binding protein that stabilizes actin filaments at the leading edge of migrating cells, and filamin-A, an actin cross-linker protein, are direct targets of AKT and play key roles in regulating cell migration [43–46]. Thus, the increased growth, motility and invasiveness that the obscurin-knockdown cells exhibit *in vitro* and *in vivo* may be direct manifestations of the activated PI3K/AKT cascade and its downstream targets.

It has been well documented that the PI3K/p85 regulatory subunit modulates the activity of the catalytic PI3K/p110 subunit [22]. Herein, we demonstrate for the first time a direct and strong interaction between giant obscurins and the PI3K/p85 regulatory subunit mediated by the PH domain of obscurins and the SH3 domain of PI3K/p85. Given the increased activation of the PI3K/AKT cascade in obscurin-depleted cells, we postulate that the direct binding of obscurins to PI3K/p85 is essential in regulating the ability of the latter to modulate the enzymatic activity of the PI3K/p110 catalytic subunit. Obscurins may mediate such an effect by topologically stabilizing the PI3K/p85 subunit in a conformation that precludes the constitutive activation of the PI3K/p110 subunit. Alternatively, the presence of a RhoGEF motif and two Ser/Thr kinase domains in obscurins, which are tandem with and proximal to the PH domain, respectively, may suggest important intra- or inter-molecular modifications, including the involvement of Rho-facilitated effects or novel phosphorylation events.

Taken together, our results demonstrate that in normal breast epithelial cells giant obscurins act upstream of the PI3K/AKT pathway contributing to its regulation via their direct association with the PI3K/p85 regulatory subunit. Conversely, loss of giant obscurins from breast cancer cells may lead to conformational and/or molecular alterations in the PI3K/p85 regulatory subunit rendering it unable to regulate the enzymatic activity of the PI3K/p110 catalytic subunit. Thus, over-activation of the PI3K/AKT pathway in obscurin-depleted breast cancer cells may, at least in part, be responsible for their increased growth, motility and invasiveness given the marked suppression of these properties following inhibition of the PI3K cascade. These findings are critical for the development of individualized chemotherapies, since obscurin-deficient breast cancer patients may substantially benefit from a targeted therapy in the form of a PI3K inhibitor rather than a generalized chemotherapy, such as the taxanes. In line with this, our earlier studies have shown that obscurin-knockdown MCF10A cells display significantly increased survival and (re)attachment capabilities in the presence of paclitaxel compared to control cells expressing scramble shRNA [10]. Thus, the single or combinatorial use of PI3K inhibitors, such as the BKM120 used in our study, which is currently in clinical trials for several types of cancer including breast cancer, would potentially be a more appropriate and effective chemotherapy for treating obscurin-deficient breast tumors.

## MATERIALS AND METHODS

### Reagents

Unless otherwise noted, all chemicals were purchased from Sigma-Aldrich (St. Louis, MO, USA). LY294002 was purchased from Cell Signaling Technology Inc (Danvers, MA, USA). BKM120 was purchased from Selleck Chemicals (Houston, TX, USA).

### Stable clones of mcf10a cells and culturing

MCF10A stable clones expressing obscurin shRNA or control shRNA plasmids were generated and maintained as described in [8].

### Antibodies

The antibodies used were as follows; rabbit polyclonal: phospho-PI3K p85 (Tyr458)/p55 (Tyr199) (4228, Cell Signaling Technology Inc, CA, USA), Twist (sc-15393, Santa Cruz Biotechnology Inc, Santa Cruz), phospho-AKT (Ser473) (4060, Cell Signaling Technology Inc), phospho-AKT (Thr308) (2965, Cell Signaling Technology Inc), phospho-GSK-3β (Ser9) (5558, Cell Signaling Technology Inc), phospho-PDK1 (Ser241) (3438, Cell Signaling Technology Inc), PDK1 (total) (3062, Cell Signaling Technology Inc), N-cadherin, (4061, Cell Signaling Technology Inc), and PI3K (total) (sc-602, Santa Cruz Biotechnology Inc, Santa Cruz, CA, USA); mouse monoclonal: β-catenin (sc-7963, Santa Cruz Biotechnology Inc), GAPDH (G7895, Sigma-Aldrich, St. Louis, MO, USA); rabbit monoclonal: E-cadherin (3195, Cell Signaling Technology Inc), vimentin (5741, Cell Signaling Technology Inc), Slug (9585, Cell Signaling Technology Inc), AKT (total) (4691, Cell Signaling Technology Inc), GSK3β (total) (12456, Cell Signaling Technology Inc), Hsp 90 (4877, Cell Signaling Technology Inc), AKT2 (3063, Cell Signaling Technology Inc), β-catenin (9582, Cell Signaling Technology Inc), PI3K/p85 (4257, Cell Signaling Technology Inc), and NH_2_-terminal obscurin [47].

### Generation of protein lysates and Western blotting

Cell lysates were prepared in radioimmuno-precipitation assay (RIPA) buffer supplemented with cocktails of protease inhibitors (Roche, Mannheim, Germany), and phosphatase inhibitors (200 nM Imidazole, 100 mM Sodium Flouride, 115 mM Sodium Molybdate, 100 mM Sodium Orthovanadate, 400 mM Sodium Tartrate Dihydrate, 100 mM β-Glycerophosphate, 100 mM Sodium Pyrophosphate, and 10 mM EGTA). Protein lysates were electrophoresed on SDS-NuPAGE gel (Thermo Fisher Scientific, Waltham, MA), transferred to nitrocellulose membranes, and probed with primary antibodies as specified in the text, and the appropriate alkaline phosphatase-conjugated secondary antibodies (Jackson ImmunoResearch Laboratories). Immunoreactive bands were visualized using Amersham^TM^ ECL^TM^ prime western blotting detection reagent (GE Healthcare Life Sciences), and densitometry was performed with Image J software. Treatment of cells followed by lysate preparation and immunoblotting analysis was repeated at least three independent times.

### Mammosphere culture

Single MCF10A cells stably transduced with obscurin shRNA were plated in ultralow attachment plates (Corning, Lowell, MA, USA) at a density of 10,000 cells/mL in 2 mL serum-free growth media (DMEM/F12 with GlutaMAX^TM^), supplemented with insulin (10 μg/mL), hydrocortisone (0.5 μg/mL), cholera toxin (100 ng/mL), epidermal growth factor (20 ng/mL), 1% penicillin-streptomycin and puromycin (1.5 μg/mL). Twenty-four hours following initial plating, cell cultures were supplemented with 2 mL serum-free growth media that contained the indicated concentration of LY294002, BKM120, or DMSO vehicle control every day for 10 days at which time point spheres were measured and those ≥ 100 μm were counted as tumor spheres.

### Cell viability and alamar blue assay

Cell viability of MCF10A obscurin-knockdown and scramble control cells treated with the specified concentrations of LY294002 and BKM120 inhibitors or vehicle DMSO for 24 h was measured using the AlamarBlue assay (Life Technologies), as earlier reported [9]. In brief, the AlamarBlue reagent was added to the cell culture at 10% v/v and incubated for 16 h at 37°C, 5% CO2. The percentage (%) of AlamarBlue reduction was determined by measuring absorbance at 550 and 620 nm. Data are presented as the percentage of AlamarBlue reduced per number of cells.

### Wound healing assay

Wound healing was measured by growing confluent MCF10A cell monolayers stably expressing obscurin shRNA in six-well tissue culture dishes (Corning). A scrape was made through the monolayer with a sterile plastic pipette tip and fresh media containing LY294002, BKM120, or DMSO vehicle control was added at the indicated concentrations. Images were taken with an inverted microscope (10X objective) at time 0 h and after a 6 h incubation period at 37°C, 5% CO_2_. Migration was expressed as the average of the difference between the measurement at time zero and 6 h obtained from 3 independent experiments.

### Invasion assay

Invasion was measured by adding 250,000 cells suspended in 0.5 mL growth media containing the specified concentration of LY294002, BKM120, or DMSO vehicle control to the upper chamber of a Matrigel-coated invasion chamber (BD Biosciences, San Jose, CA). The lower chamber contained growth media supplemented with 10% FBS. The inserts were incubated at 37°C, 5% CO_2_ for 16 h. At the end of the 16 h incubation period, the cells that had invaded in the lower chamber were fixed and stained with 0.5% crystal violet in 20% methanol. The number of invaded cells was quantified by counting at least 6 random fields from 3 independent experiments under an inverted light microscope (Olympus IX51) with a 10X objective.

### Microchannel seeding and single cell migration

The microchannel device was fabricated by standard lithography and coated with 20 μg/mL collagen type I (BD Biosciences, San Jose, CA), as previously described [40, 48–51]. Cells were trypsinized, resuspended in serum-containing media to neutralize the trypsin, and subsequently washed in serum-free media. A suspension of 5 × 10^4^ cells was added to the inlet port, and cells were transported along the seeding channel by pressure-driven flow. Within 5 min, the cell suspension was removed and replaced with 100 μL of serum-free media containing the specified concentrations of LY294002, BKM120, or DMSO vehicle control. Serum-containing media with the indicated concentrations of LY294002, BKM120, or DMSO was added to the top-most inlet port, thus forming a chemoattractant gradient. Chambers were placed in an enclosed, humidified microscope stage at 5% CO2 and 37°C (TIZ, Tokai Hit Co., Japan or Okolab, Italy). Phase contrast time-lapse images were captured at 20-min intervals for up to 17 h on an inverted Nikon microscope (10X objective) at multiple stage positions via stage automation (Nikon Elements, Nikon, Japan). Cell x,y position within the microchannel was identified as the midpoint between the poles of the cell body and tracked as a function of time using ImageJ (NIH, Bethesda, MD) and the MTrackJ plugin for up to 8 h [52]. Cells in the microchannels for less than 1 h were not tracked. Tracks were discontinued if the cell left the microchannel. Dividing cells were not tracked. Cell velocity and chemotactic index were computed using a custom-written Matlab program (The MathWorks, Natick, MA). Instantaneous cell velocity was calculated by dividing each interval displacement by the time interval (20 min), and the mean velocity for a given cell was computed by averaging instantaneous velocities for all time intervals. Chemotactic index was calculated by dividing the end-to-end displacement by the total path length of the cell. The reported velocity and chemotactic index for each condition is the mean of the pooled cells from at least 3 independent experiments.

### Co-immunoprecipitation assays

Co-immunoprecipitation experiments were performed with protein lysates prepared in RIPA buffer from parental MCF10A cells, according to [53]. In brief, 100 μL of protein A/G beads slurry (Thermo Fisher) were incubated with 5 μg of NH_2_-terminal obscurin antibody or mouse IgG (Jackson ImmunoResearch Laboratories Inc, West Grove, PA) at 4°C overnight in PBS. The antibody-bound beads were then incubated with 1 mg of MCF10A protein lysates at 4°C overnight with gentle rocking. Samples were washed 5x with PTA (PBS containing 0.5% Tween-20), solubilized in 60 μL 2x SDS-PAGE sample buffer, and heated at 70°C for 20 min before they were separated by SDS-PAGE and transferred to nitrocellulose membranes. Blots were probed with the indicated primary antibodies and the appropriate alkaline phosphatase-conjugated secondary antibodies (Jackson ImmunoResearch Laboratories). Immunoreactive bands were visualized with the Tropix chemiluminescence detection kit (Applied Biosystems).

### Production of recombinant proteins and pull down assays

The Pleckstrin Homology (PH) domain of obscurin and the Src Homology 3 (SH3) domain of the regulatory subunit of PI3K were produced as glutathione-S-transferase (GST, PGEX vector; GE Healthcare Life Sciences) and His (pET-30a(+) vector; EMD Millipore, Billerica, MA) tagged proteins, respectively, according to the manufacturers’ instructions and our established methods [54]. Human cDNA from normal breast tissue was purchased from a commercial source (Origene, Atlanta, GA), and the following sets of primers were used for amplification of the obscurin-PH and the PI3K/p85-SH3 domains: Sense-Obsc-PH: 5′ CCCATCCGCCAGGGC 3′ and Antisense-Obsc-PH: 5′ AGGCAGGGCCAGACG 3′ (NM_001098623.2), and Sense-PI3K/p85-SH3: 5′ CCTGAGGGCTTCCAG 3′ and Antisense-PI3K-SH3: 5′ TCAGGGCCCCAGGAA 3′ (NM-005027). GST- and HIS-tagged recombinant proteins were expressed by induction with 0.5 mM isopropyl β-D-thioglucopyranoside (IPTG) for 3 h and purified by affinity chromatography on glutathione-Sepharose (Thermo Fisher Scientific) or Talon cobalt (Thermo Fisher Scientific) columns, respectively, following the manufacturers’ instructions.

GST-pull down assays were performed as described before [54, 55]. Equivalent amounts of control GST-protein and GST-obscurin-PH were bound to glutathione-Sepharose beads and incubated with 3 μg/mL of HIS-tagged PI3K/p85-SH3 protein overnight at 4°C in pull-down buffer (50 mM Tris, pH 7.5, 120 mM NaCl, 10 mM NaN3, 2 mM DTT, and 0.5% Tween). At the end of the incubation period, beads were washed five times with wash buffer (PBS with 10 mM NaN3 and 0.1% Tween), and bound proteins were eluted with 2× LDS buffer (Invitrogen), followed by boiling at 95°C for 10 min and separation on a 4–12% bis-Tris gel. A HIS6-antibody (sc-803, Santa Cruz Biotechnology) was used for detection of immunoreactive bands with the Tropix chemiluminescence detection kit (Applied Biosystems).

### Kinetic analysis of obscurin-PH binding to PI3K/p85-SH3 using surface plasmon resonance

Surface plasmon resonance was performed using a BiaCore 3000 instrument, as previously described [6, 54, 56]. In particular, the GST-obscurin-PH domain was used as ligand and was immobilized on a carboxymethyl-dextran sensor (CM5) chip, while the HIS-PI3K-p85/SH3 domain was used as analyte at varying concentrations ranging from 8–250 nM. The flow rate for analyte injection was 20 μl/min. For each analyte concentration, association was measured for 180 sec and dissociation was measured over another 180 sec. Data were evaluated with the 1:1 Langmuir model and the Heterogeneous Ligand model (BIAevaluation Software 3.1, Biacore). The association (*k*_a_, M^−1^ s^−1^) and dissociation (*k*_d_, s^−1^) rate constants as well as the association (*K*_A_, M^−1^) and dissociation (*K*_D_, M) equilibrium constants were determined.

### Reproducibility and statistics

All experiments were performed in triplicates at a minimum of three times, and data are presented as mean values of independent measurements ± Standard Deviation (SD). Statistical significance was assessed using Student's *t*-test or Mann-Whitney test.

## SUPPLEMENTARY MATERIALS FIGURES AND VIDEOS






