# Supplementary material for: Giant obscurins regulate the PI3K cascade in breast epithelial cells via direct binding to the PI3K/p85 regulatory subunit
Source: Oncotarget. 2016 Jun 13;7(29):45414–28. doi: 10.18632/oncotarget.9985 (PMC5216731; doi:10.18632/oncotarget.9985)
Supplement: Supplementary file 1 [file oncotarget-07-45414-s001.pdf]

Giant obscurins regulate the PI3K cascade in breast epithelial cells via direct binding to the PI3K/p85 regulatory subunit

Supplementary Materials

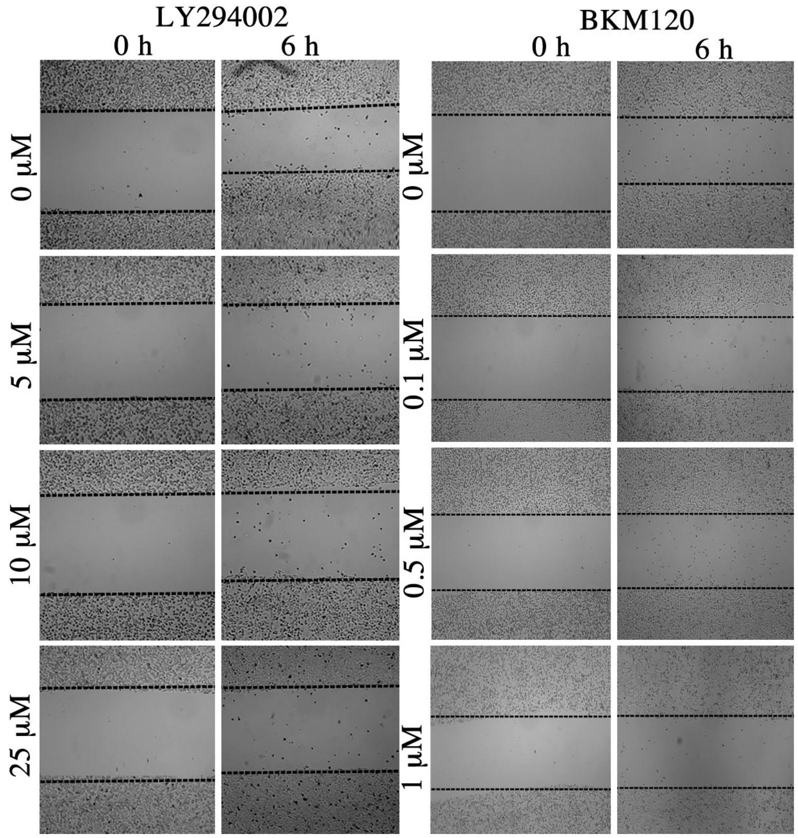

Supplementary Figure S1: Representative images of confluent monolayers of obscurin-knockdown MCF10A cells at 0 and 6 h post-wounding in the presence of vehicle DMSO (top panels) or LY294002 (5–25 µM) and BKM120 (0.1–1 µM) inhibitors. Inhibitor treatment decreases the ability of cells to close the wound in a dose-dependent manner.

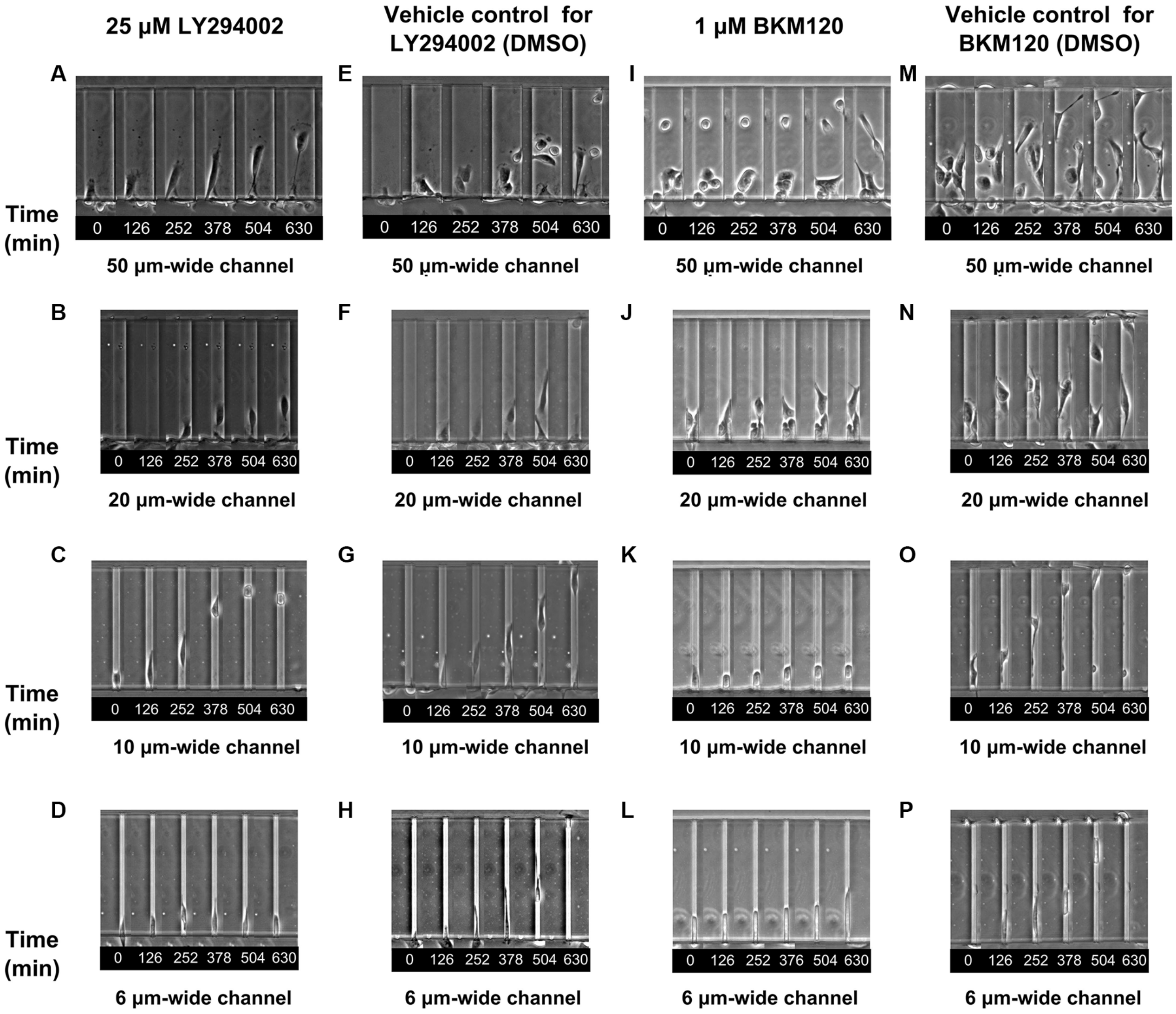

Supplementary Figure S2: Suppression of PI3K signaling inhibits the chemotactic migration of MCF10A obscurin-knockdown cells through microfabricated microchannels. MCF10A obscurin-knockdown cells were treated with 25 µM LY294002 (**A**–**D**), 1 µM BKM120 (**I**–**L**), or vehicle DMSO (**E**–**H** and **M**–**P**). Microchannels were fabricated in PDMS and were 10 µm tall. Channels width was set at 50, 20, 10, or 6 µm. Timelapse images of cells migrating upon treatment with the respective inhibitors and in the mirochannels of the indicated widths are shown. Cells migrated up a 5% horse serum gradient.

Supplementary Video S1: MCF10A obscurin-knockdown cells were seeded in PDMS microchannel devices and treated with 25 µM LY294002 or the appropriate vehicle control (DMSO). Cells migrated through 10 µm-tall microchannels of 6, 10, 20, or 50 µm width up a chemotactic stimulus. Migrating cells were imaged using phase contrast microscopy. Video shows images taken 10.5 min apart and is replayed at 7 frames per second; scale bar = 50 µm.

Supplementary Video S2: MCF10A obscurin-knockdown cells were seeded in PDMS microchannel devices and treated with 1 µM BKM120 or the appropriate vehicle control (DMSO). Cells migrated through 10 µm-tall microchannels of 6, 10, 20, or 50 µm width up a chemotactic stimulus. Migrating cells were imaged using phase contrast microscopy. Video shows images taken 10.5 min apart and is replayed at 7 frames per second; scale bar = 50 µm.
